# Design Principles of the Rotary Type 9 Secretion System

**DOI:** 10.3389/fmicb.2022.845563

**Published:** 2022-05-10

**Authors:** Abhishek Trivedi, Jitendrapuri Gosai, Daisuke Nakane, Abhishek Shrivastava

**Affiliations:** ^1^School of Life Sciences, Arizona State University, Tempe, AZ, United States; ^2^Biodesign Center for Fundamental and Applied Microbiomics, Arizona State University, Tempe, AZ, United States; ^3^Center for Biological Physics, Arizona State University, Tempe, AZ, United States; ^4^Department of Engineering Science, The University of Electro-Communications, Tokyo, Japan

**Keywords:** bacterial motility, T9SS, gliding motility, bacterial swarming, Flavobacteria

## Abstract

The F_o_ ATP synthase, the bacterial flagellar motor, and the bacterial type 9 secretion system (T9SS) are the three known proton motive force driven biological rotary motors. In this review, we summarize the current information on the nuts and bolts of T9SS. Torque generation by T9SS, its role in gliding motility of bacteria, and the mechanism *via* which a T9SS-driven swarm shapes the microbiota are discussed. The knowledge gaps in our current understanding of the T9SS machinery are outlined.

## The Diversity of Bacterial Protein Secretion Systems

**FIGURE 1:**
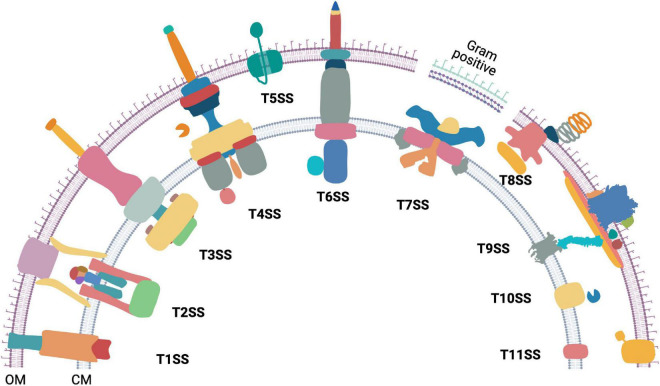
The diversity of bacterial protein secretion systems. Graphical representation of the currently known bacterial protein secretion systems ranging from Type 1 to Type 11 and their localization in the outer (OM) and cytoplasmic membrane (CM). Except T7SS (Gram positive), all the above shown secretion systems are found in Gram negative bacteria. Occurrence of T7SS mediated protein secretion is found within some Gram positive members of the phylum Actinobacteria that have an outer lipid layer.

The hydrophobic lipid bilayer of the bacterial cell membrane stops proteins from diffusing outside the cell. However, physiological processes such as the degradation and uptake of complex extracellular nutrition sources, adhesion to biotic and abiotic surfaces, colonization, motility, virulence, and interbacterial antagonism require transport of proteins across membranes. Protein secretion systems serve this purpose and form a pore that provides a gateway to a select group of proteins. Thus far, eleven bacterial protein secretion systems have been discovered (Figure 1). Additionally, the Sec and Tat transporters (Kudva et al., 2013), Chaperon-usher pathway (Waksman and Hultgren, 2009), the YidC insertase (Hennon et al., 2015), Sortases (Spirig et al., 2011), and a TonB-dependent machinery (Gómez-Santos et al., 2019) are also involved in bacterial protein export. Majority of this review is focused on the bacterial Type 9 Secretion System (T9SS), its mechanism of rotation, gliding motility, and collective behavior. However, without an appreciation of the diversity of bacterial secretion machineries, the beauty of T9SS can be lost on the reader. Hence, we initiate this review with a brief and up-to date overview of bacterial protein secretion machineries.

Type 1 to Type 11 bacterial secretion systems are known to facilitate extracellular transport of proteins. Some bacterial secretion systems work in conjunction with either the Sec or the Tat transporters, whereas others work independently. The bacterial Sec protein export pathway allows transport of unfolded proteins through the cytoplasmic membrane (Papanikou et al., 2007; Kusters and Driessen, 2011; Denks et al., 2014) while the Twin Arginine translocation (Tat) system allows transport of folded proteins across the cytoplasmic membrane (Palmer and Berks, 2012). Interestingly, some protein secretion systems are associated with machineries that power bacterial motility. Examples include the Type 2 Secretion System (T2SS) associated type IV pilus (Craig et al., 2019), the Type 3 Secretion System (T3SS) associated with the flagellar motor (Abrusci et al., 2013), and the T9SS that is integral to gliding motility of Bacteroidetes (Sato et al., 2010; Shrivastava et al., 2013; Zhu and McBride, 2014; Kita et al., 2016; Lauber et al., 2018; Shrivastava and Berg, 2020; Hennell James et al., 2021).

The Type 1 Secretion System (T1SS) is comprised of an ABC transporter-like protein complex spanning the cytoplasmic membrane. It is coupled with a viral fusion protein and a β-barrel channel that spans the outer membrane. T1SS enables secretion of unfolded protein substrates from the cytoplasm to the extracellular milieu (Spitz et al., 2019). The T2SS secretes proteins that are already exported to the periplasm *via* Sec or Tat pathway. The multiprotein piston-like pseudopilus component of the T2SS pushes its folded protein substrates out of the outer membrane secretin in a process powered by ATP hydrolysis (Korotkov and Sandkvist, 2019; Naskar et al., 2021). The T3SS forms an injectisome and transports proteins from the cytosol either to the extracellular milieu or across the plasma membrane of a eukaryotic host cell (Portaliou et al., 2016). The core of both the bacterial flagellar apparatus and the injectisome have a T3SS. While there is some debate on the evolution of injectisome and the flagellum, it has been proposed that the flagellum is the ancestor of the injectisome-T3SS complex (Diepold and Armitage, 2015). The Type 4 Secretion System (T4SS) is found in both eubacteria and archaea. It exports both proteins and DNA from the cytosol of T4SS containing bacteria to either a prokaryotic or a eukaryotic host cell (Li et al., 2019). Additionally, a class of T4SS is also employed by bacteria to take up extracellular DNA (Hofreuter et al., 2001; Li et al., 2019). The Type 5 Secretion System (T5SS), also known as autotransporter, contains a single protein that spans the Gram negative bacterial outer membrane. The translocator domain of the T5SS protein is a β-barrel. In most cases, its secretory substrate (passenger domain) is a polypeptide chain contiguous to the translocator domain (Fan et al., 2016; Meuskens et al., 2019). The Type 6 Secretion System (T6SS) is formed by the association of homologs of T4SS components and bacteriophage contractile tails. It is widely employed to inject effector proteins from the cytoplasm directly across other bacterial or eukaryotic host membranes (Cianfanelli et al., 2016; Coulthurst, 2019). The Type 7 Secretion System (T7SS) is found in Gram positive bacteria of the genus *Mycobacterium* and other members of the phylum Actinobacteria. Five paralogous *esx* loci are found in Mycobacteria and a recent high resolution Cryo-EM structure of an Esx-5 paralog revealed the formation of an inner membrane pore (Bunduc et al., 2020; Beckham et al., 2021; Rivera-Calzada et al., 2021). The Type 8 Secretion System (T8SS), widely known as the curli biogenesis pathway spans the outer membrane of Gram-negative bacteria. It secretes monomeric curli particles and enables their extracellular assembly and nucleation (Van Gerven et al., 2015; Bhoite et al., 2019). The Type 9 Secretion System (T9SS) spans both membranes and is found in the Gram negative Fibrobacteres-Chlorobi-Bacteroidetes superphylum. The substrates are delivered to the periplasm by Sec transport pathway and T9SS secretes them across the outer membrane. The T9SS contains a motor that drives secretion. This is one of the only three known biological rotary motors driven by a proton motive force (pmf) and is the focus of this review. The components of Type 10 Secretion System (T10SS) comprise of holins and peptidoglycan modifying enzymes. T10SS secretes hydrolytic enzymes and toxins (Palmer et al., 2021). Recently, the presence of a Type 11 Secretion System (T11SS) was proposed. It comprises of DUF560 family proteins and it transports periplasmic cargo across the outer membrane (Grossman et al., 2021).

## A Brief History of Type 9 Secretion System

Proteins that are now known to form the core of the bacterial type 9 secretion system (T9SS) were discovered around similar timelines in two model organisms *Flavobacterium johnsoniae* and *Porphyromonas gingivalis*. Transposon mutagenesis led to the discovery that *F. johnsoniae* GldK, GldL, GldM, and GldN are required for motility and chitin utilization (Braun et al., 2005). Similarly, it was identified that *P. gingivalis* PorT (Sato et al., 2005) and SprA/Sov are required for the transport of gingipain proteases (Saiki and Konishi, 2010). Later, it was shown that PorT of *F. johnsoniae* is also required for the secretion of chitinase and that GldK, GldL, GldM, and GldN form a multi-protein complex. Hence, it was inferred that these set of proteins form a secretion system that was initially named as the Por secretion system (PorSS) (Sato et al., 2010). It was later found that these proteins are prevalent in the Bacteroidetes phylum (McBride and Zhu, 2013; Shrivastava, 2013) and they are required for secretion of multiple classes of proteins. Because the components of the T9SS are not similar to those of other secretion systems, it was renamed as T9SS (Shrivastava et al., 2013). In recent years, the T9SS field has moved forward by leaps and bounds. We now know that T9SS forms a rotary machinery that enables gliding motility (Shrivastava et al., 2015) and we know the identity and structure of the ion channel that powers rotation (Shrivastava et al., 2015; Hennell James et al., 2021). Additionally, structures of several T9SS proteins have recently been resolved (Gorasia et al., 2016; Lauber et al., 2018; Leone et al., 2018; Trinh et al., 2020; Hennell James et al., 2021). T9SS secretes many enzymes, adhesins, and virulence factors. The signatures of T9SS secreted proteins have recently been characterized (Sato et al., 2013; de Diego et al., 2016; Kulkarni et al., 2017, 2019).

## Diversity of Substrates Secreted by Type 9 Secretion System

Type 9 secretion system driven protein export occurs in conjunction with the Sec transport machinery. T9SS substrates have a N-terminal Sec signal peptide and a C-terminal T9SS signal sequence (CTD). *Via* the Sec system, T9SS substrates reach the periplasm and the N-terminal Sec signal peptide is cleaved. T9SS CTD containing periplasmic proteins are exported either to the extracellular milieu or they attach to the outer membrane (OM) (Slakeski et al., 2011; Kulkarni et al., 2017, 2019). T9SS secreted extracellular proteins lose their CTD (Sato et al., 2013) and it is unclear whether CTD cleavage occurs during or after transport. The T9SS CTD is divided into two protein domain families namely TIGR04183 (Type A CTD) and TIGR04131 (Type B CTD). Type A CTD have seven β strands and lg-like folds which are thought to interact with the structural components of T9SS (de Diego et al., 2016; Lasica et al., 2016). Super folder GFP (sfGFP) fused with T9SS CTD and N-terminal Sec signal peptide is secreted by T9SS (Kulkarni et al., 2017, 2019) which further confirms that substrate recognition by T9SS only occurs due to the CTD. Additionally, a T9SS secreted chitinase possesses a CTD different from both Type A and Type B CTD (Kharade and McBride, 2014). We found that on InterPro (Blum et al., 2021) 179,000 different bacterial proteins are annotated to have TIGR04183 domain and 28,000 different proteins are annotated to have TIGR04131 domain. Many of T9SS encoding proteomes belong to bacteria of the phylum Bacteroidetes. The environmental microbe *F. johnsoniae* UW101 encodes 40 Type A and 12 Type B CTD containing proteins. These include the motility adhesins SprB (Nelson et al., 2008) and RemA (Shrivastava et al., 2012). Additionally, multiple enzymes including a chitinase are secreted by the T9SS of *F. johnsoniae* (Kharade and McBride, 2014, 2015). SprB is one of the largest known adhesin protein (669 kDa) and cells lacking SprB are severely deficient for gliding motility (Nelson et al., 2008). SprB moves spirally on the bacterial cell-surface (Nakane et al., 2013; Shrivastava et al., 2016) and is driven by pmf (Nakane et al., 2013). Adhesion of SprB to an external substratum enables screw-like motion of the rod-shaped *F. johnsoniae* (Shrivastava et al., 2016). The human oral microbe *P. gingivalis* W83 encodes 17 Type A CTD-containing proteins (including the virulent gingipain proteases) and one Type B CTD-containing protein. In contrast, another prominent human oral microbe *Capnocytophaga ochracea* ATCC 27872 encodes 2 Type A CTD containing proteins and 8 Type B CTD containing proteins.

**FIGURE 2:**
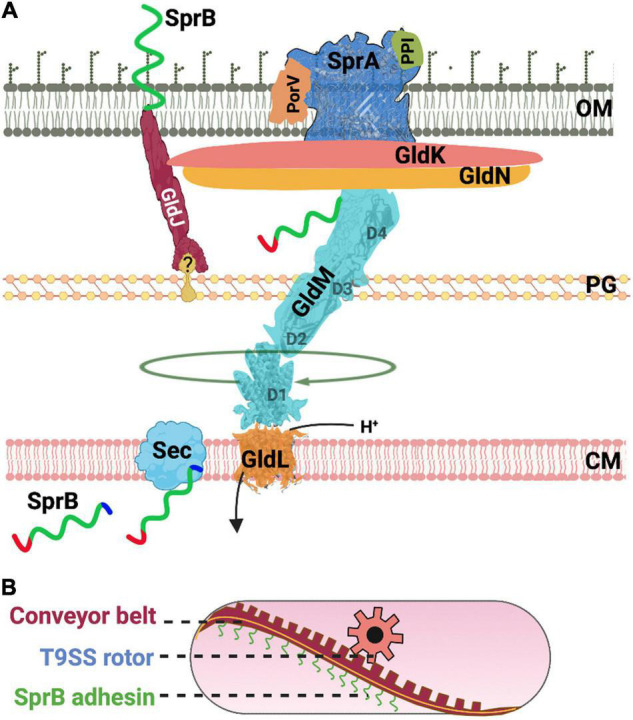
Structure and function of the T9SS. **(A)** A cartoon of the nuts and bolts of the T9SS motor that drives protein secretion and gliding motility. T9SS substrates (SprB shown as an example) are transported to the periplasm *via* the Sec transport pathway. The CTD of T9SS substrates is cleaved during transport. A recent model suggests that the proton channel GldL powers the rotation of T9SS. **(B)** A cartoon of the molecular rack and pinion machinery that drives gliding motility. A model based on recent data suggests that the rotary T9SS pinion drives a cell-surface conveyor belt (rack). Cell-surface adhesins such as SprB are secreted by T9SS and are loaded onto the conveyor belt. Interaction of SprB with an external substratum results in screw-like gliding motility of the bacterial cell.

Many of the shared T9SS components identified in *F. johnsoniae* and *P. gingivalis* were given different names (e.g., Gld vs. Por). These nuts and bolts of T9SS enable both gliding motility and protein secretion (Figure 2A). Below, we compile information on T9SS proteins from both model organisms *F. johnsoniae* and *P. gingivalis*.

## The Nuts and Bolts That Form the Core of Rotary Type 9 Secretion System

*GldK/PorK* (Fjoh_1853/PGN_1676) is attached to the outer membrane *via* a lipid anchor and is localized in the periplasmic space (Shrivastava et al., 2013; Vincent et al., 2017). GldK/PorK of *P. gingivalis* forms a ring that has an outer diameter of 50 nm and a pore of 35 nm. This ring comprises 32–36 GldK/PorK monomers (Gorasia et al., 2016, Gorasia D. et al., 2020).

*GldN/PorN* (Fjoh_1856/PGN_1673) forms a ring and is associated with GldK/PorK in a 1:1 stoichiometry (Gorasia et al., 2016, Gorasia D. et al., 2020). GldN/PorN is localized in the periplasm. *Flavobacterium johnsoniae* has a paralog of GldN/PorN called as GldO. The two proteins are partially redundant. In the genomic context, *gldO* of *F. johnsoniae* is located next to *gldN* (Rhodes et al., 2010).

*GldL/PorL* (Fjoh_1854/PGN_1675) is a cytoplasmic membrane protein (Shrivastava, 2013) and is found near the axis of rotation of a tethered cell (Shrivastava and Berg, 2020). *Flavobacterium johnsoniae* cells containing amino acid substitution of protonatable residues of GldL/PorL (E49Q and E49D) are completely deficient for secretion (Hennell James et al., 2021). Additionally, secretion is reduced for strains that contain GldL/PorL Y13A and K27A substitutions. This suggests that GldL/PorL is an ion channel that generates pmf driven power stroke for the rotation of T9SS (Hennell James et al., 2021). Aspartate and glutamate residues of ion channels are known to play important roles in the generation of pmf (Zhou et al., 1998; Vorburger et al., 2009). GldL/PorL has two glutamates, one is in the transmembrane helix 2 (TMH2) (E49) and the other (E59) is located between TMH2 and cytoplasmic domain. Cells with GldL/PorL E49A substitution are unable to secrete SprB and fail to attach to a glass surface. Cells with GldL/PorL E59A substitution secrete SprB to the surface yet they are non-motile (Vincent et al., 2021).

*GldM/PorM* (Fjoh_1855/PGN_1674) interacts with GldL/PorL to form an asymmetric complex with two subunits of GldL/PorL surrounded by five subunits of GldM/PorM. It has one transmembrane domain that contains a protonable residue. Y17A substitution in the transmembrane domain of GldM/PorM resulted in reduced secretion. GldM/PorM has four periplasmic domains D1–D4. X-ray crystallography of GldM/PorM showed a hinge between D2 and D3 (Leone et al., 2018) whereas cryo-EM structural determination of truncated GldM/PorM in complex with GldL/PorL showed a bend between D1 and D2 (Hennell James et al., 2021).

## The Mechanism of Rotation and Comparison of Type 9 Secretion System With the Bacterial Flagellar Motor

Similar to the flagellar FlgH-FlgI LP ring that forms a molecular bushing, GldK/PorK-N rings of T9SS localize in the outer membrane and periplasmic region. Like GldK/PorK, FlgH is a lipoprotein and like GldN/PorN, FlgI is a periplasmic protein. The LP ring of the flagellar motor interacts with the distal end of the driveshaft (rod), supports stable rotation of the rod, and reduces friction (Yamaguchi et al., 2021). The GldK/PorK-N ring may play a similar role in T9SS rotation. If it does, this implies that T9SS might have a currently undiscovered driveshaft. Alternatively, the mechanism of torque generation could be unique and the rotary component (Gld/PorK-N ring) could be placed on the periplasmic face of the outer membrane of T9SS containing bacteria.

Based on structural data, a model was recently presented where due to asymmetry, GldL/PorL enables rotation of GldM/PorM (Hennell James et al., 2021). The only experimental evidence for rotation of T9SS is from tethered cell analysis (Shrivastava et al., 2015) and it depicts rotation of an outer membrane protein. How does rotary GldM/PorM cross the peptidoglycan barrier and transmit rotation to an outer membrane protein? In one scenario, it might be possible that GldM/PorM might pass through a pore in the peptidoglycan. This pore could be formed by a protein that has a structure like the P ring bushing of the flagellar motor. Given the current structural data, the ring shaped GldN/PorN appears to be a candidate for the bushing. GldM/PorM might be the driveshaft that passes the peptidoglycan *via* the GldN/PorN bushing, and it can induce rotation of the outer membrane associated GldK/PorK ring. However, one caveat to this model is that GldN/PorN ring is shown to directly interact with the GldK/PorK ring (Gorasia et al., 2016). It is possible that the linkage between GldK/PorK and GldN/PorN is weak and transient. Hence, GldM/PorM driven GldK/PorK might rotate relative to the static GldN/PorN bushing. There is also a possibility that GldN/PorN might not form the bushing and it rotates along with GldK/PorK ring. This scenario implies that another protein might from the pore that allows passage of GldM/PorM through the peptidoglycan. Future structural and biochemical data will help fill the current knowledge gaps regarding the mechanism of rotation of T9SS.

*PorE* (Multiple homologs in *F. johnsoniae*/PGN_1296) is a periplasmic protein that is anchored to inner leaflet of outer membrane (Heath et al., 2016). Sequence analysis and modeling suggest that PGN_1296 has four domains (i) a tetratricopeptide repeat domain (TPR, residues 25–149); (ii) a β–propeller domain (WD40, residues 167–435); (iii) a carboxypeptidase regulatory domain-like fold (CRD, residues 441–527) and (iv) an OmpA_C-like putative peptidoglycan-binding domain (PBD) (residues 534–668). The OmpA_C (PBD) structure of PorE consists of a three-stranded β-sheet (β1–3) and five α-helices (α1–5) (Trinh et al., 2020).

The MotA–MotB stator units of the flagellar motor are attached to the cell wall by a peptidoglycan binding domain of MotA. Neither GldL/PorL nor GldM/PorM contain a detectable peptidoglycan binding domain. For the Gld/PorLM complex to function as a stator unit, attachment to a rigid part of the cell is necessary. This might be achieved by the PBD of PorE. However, there is no direct evidence for interaction between PorE and Gld/PorLM and whether PorE enables T9SS anchoring remains to be seen. *P. gingivalis* cells lacking PorE have reduced T9SS activity (Heath et al., 2016). The Role of PorE homolog of *F. johnsoniae* is unknown. There are multiple homologs of PorE in *F. johnsoniae*. Future experimental evidence might clear our understanding of anchoring of GldL/PorL. In an alternate scenario, it is possible that PorE might not interact with core T9SS proteins and GldL/PorL might be anchored *via* a cytoskeleton protein.

*SprF/PorP* (Multiple homologs in *F. johnsoniae*/PGN_1677) of *P. gingivalis* has been reported to interact individually with PorE, Gld/PorK and GldM/PorM (Gorasia D. et al., 2020) and could be the connection between T9SS and the peptidoglycan bound PorE. However, if this scenario is correct, neither GldK/PorK ring or GldM/PorM can rotate. It might imply that like LP rings of the flagellar motor, both GldK/PorK and GldN/PorN act as bushings. Alternatively, as discussed below, the interaction of SprF/PorP with PorE might not have any role in motility and it might only occur during the process of protein secretion.

SprF/PorP is required for secretion of TIGR04131 domain containing proteins. In the genomic context, a cell often has multiple copies of SprF/PorP and genes encoding SprF/PorP are often localized next to genes encoding TIGR04131 domain containing proteins (Kulkarni et al., 2019). As an example, one *F. johnsoniae* SprF encoding gene (Fjoh_0978) is localized downstream of the mobile cell-surface adhesin SprB encoding gene (Fjoh_0979). SprF/PorP is required for secretion of SprB, and it might be possible that SprF interacts with SprB during transport. If correct, this might imply that the interaction of SprF/PorP with GldK/PorK and GldM/PorM happens solely during transport and that it might help the T9SS pore recognize TIGR04131 containing substrates.

*SprA/Sov* (Fjoh_1653/PGN_0832) is required for secretion SprB, RemA, chitinase, and gingipains (Saiki and Konishi, 2007; Shrivastava et al., 2013). It forms the largest single polypeptide outer membrane ß-barrel (36 strand) known and acts as the protein translocon of the T9SS. SprA is found in two states one with PorV and the other with a plug protein. In both the states, SprA is bound to a lipoprotein Peptidyl-prolyl cis-trans isomerase (PPI). SprA forms a transmembrane β-barrel structure which rises about 20 Å above the membrane on the cell-surface. Two folded inserts are present between β-barrel strand 7–8 and 11–12. They form a 50 Å high cap structure that seals the extracellular end of the barrel. The cap insert consist of two domains with a β-sheet structure and the first domain surrounding the second domain from both the sides (Lauber et al., 2018).

*PorV* (Fjoh_1555/PGN_0023) is bound to the outer membrane pore of SprA and acts as a gate. There are highly conserved packing interactions between PorV and the lateral opening of SprA. When in complex with SprA, the PorV barrel is tilted by 25°. While in a free state, it is proposed that PorV adopts a more vertical position in the membrane bilayer (Lauber et al., 2018). *F. johnsoniae* cells lacking PorV are deficient in secretion of the motility adhesin RemA, the chitinase ChiA, and many other proteins. However, cells lacking PorV have the ability to secrete SprB hence they form spreading colonies on agar (Kharade and McBride, 2015).

Peptidyl-prolyl *cis-trans* isomerase (PPI: Fjoh_4997/PGN_0742/PGN_0744) binds to the lateral face of SprA opposite to PorV. PPIases are chaperones which catalyze the cis/trans isomerization of proline and act as a regulatory switch during protein folding (Dunyak and Gestwicki, 2016). Deletion of this PPI has no notable effect on T9SS function or gliding motility (Lauber et al., 2018). However, another predicted PPI, GldI (discussed later), is essential for motility.

*Plug* (Fjoh_1759/putative PGN_0144) can be bound to SprA ß-barrel when SprA is not in a complex with PorV. The plug protein mutant neither has gliding defect nor is defective in T9SS secretion. When PorV is not bound to SprA, the plug prevents leakage of small molecules through the SprA pore (Lauber et al., 2018).

*SprT/PorT* (Fjoh_1466/PGN_0778) is an outer membrane protein (Nguyen et al., 2009). Weak activity of gingipains Kgp and RgpA are observed in lysates and culture supernatants of *P. gingivalis* cells that lack PorT (Sato et al., 2005). *F. johnsoniae* cells lacking SprT form non-spreading colonies on agar. They fail to secrete SprB to the cell surface, and are deficient in chitin utilization (Sato et al., 2010).

*SprE/PorW* (Fjoh_1051/PGN_1877) is required for the secretion of an array of T9SS proteins (Gorasia D. G. et al., 2020). *F. johnsoniae* cells lacking SprE exhibit very little motility and are deficient in chitin utilization (Rhodes et al., 2011). It is reported that PorW of *P. gingivalis* acts as a bridge between Sov/SprA translocon and PorK/N rings (Gorasia et al., 2022).

## Additional Proteins Required for Gliding Motility

It is proposed that a molecular rack and pinion assembly enables gliding motility of Bacteroidetes (Shrivastava and Berg, 2020). Here, T9SS rotary motor is the pinion that pushes a conveyor belt on the cell-surface (Figure 2B). As described above, the identity of proteins that form T9SS is now well-known, however, the nature of proteins that form the rack or the conveyor belt are mysterious. Comparative genomic analysis has shown that non-motile Bacteroidetes lack several accessory Gld proteins. It is possible that the additional Gld proteins help in the formation of the rack or the conveyor belt (rack) which is only found in motile Bacteroidetes. The localization and function of the additional Gld proteins are described below.

*GldJ* (Fjoh_1557) is a lipoprotein that appears to form helical tracks on the cell-surface (Braun and McBride, 2005) and it might be one of the central components of the gliding machinery. GldJ is required for the stability of GldK (Johnston et al., 2018). However, *F. johnsoniae* cells lacking GldK/PorK, GldL/PorL, GldM/PorM, and GldN/PorN have wild-type like levels of GldJ (Braun et al., 2005). Cells lacking either 8 or 13 C-terminal amino acids (AA) of GldJ have functional T9SS, however, cells lacking C-terminal 13 AA of GldJ are completely non-motile and cells lacking C-terminal 8 AA of GldJ exhibit very little motility. This is the first instance where the motility phenotype is completely separated from T9SS secretion (Johnston et al., 2018). GldJ has 30% sequence similarity with *F. johnsoniae* GldK/PorK. It is a homolog of a sulfatase activating enzyme (SUMF1) but it lacks the active site of SUMF1 (Braun and McBride, 2005).

*GldA* (Fjoh_1516) belongs to the ATP binding cassette family of transport proteins (ABC transporter proteins) essential for gliding motility. GldA has Walker “A” and “B” motifs, which are characteristics of an ATP hydrolyzing domain of an ABC transporter (Agarwal et al., 1997). Furthermore, GldA point mutations in ABC transporter putative active site G40R result in reduction of cellular levels of GldJ and GldK/PorK. Similarly, a point mutation in ABC transporter signature sequence LSKGYRQR (site of point mutation underlined) shows that a functional GldA is required for the stability of both GldJ and GldK/PorK (Braun et al., 2005; Johnston et al., 2018).

*GldB* (Fjoh_1793) is a lipoprotein essential for gliding motility and cells lacking GldB have a defective T9SS (Hunnicutt and McBride, 2000). GldB is required for the stability of both GldJ and GldK/PorK (Braun and McBride, 2005; Johnston et al., 2018).

*GldD* (Fjoh_1540) is a lipoprotein essential for gliding motility (Hunnicutt and McBride, 2001; McBride et al., 2003). Furthermore, *F. johnsoniae* cells lacking GldD do not propel latex beads. GldD is required for the stability of both GldJ and GldK/PorK (Braun and McBride, 2005; Johnston et al., 2018).

*GldF* (Fjoh_2722) is essential for gliding motility (Hunnicutt et al., 2002). Both GldF and GldG are required for membrane localization of GldA (Agarwal et al., 1997; Hunnicutt et al., 2002). GldF is required for the stability of GldK/PorK (Johnston et al., 2018).

*GldG* (Fjoh_2721). Cells lacking GldG are non-motile (Hunnicutt et al., 2002). GldG is predicted to be a transmembrane protein that interacts with GldA to form an ABC transporter (Agarwal et al., 1997; Hunnicutt et al., 2002).

*GldH* (Fjoh_0890) is a lipoprotein essential for gliding motility. Cell lacking GldH do not propel latex beads and are unable to utilize chitin (McBride et al., 2003). GldH is required for the stability of GldJ and GldK/PorK (Braun and McBride, 2005; Johnston et al., 2018).

*GldI* (Fjoh_2369) mutants form non-spreading colonies on agar. GldI has a PPI domain (McBride and Braun, 2004). Cells lacking GldI do not propel latex beads and are unable to utilize chitin. GldI is required for the stability of GldJ and GldK/PorK (Braun and McBride, 2005; Johnston et al., 2018).

## Accessory Proteins Not Required for Gliding Motility

Evolutionary forces can drive modifications in components of biological machineries from different model organisms. As examples, the shape of bacterial flagellar motor varies amongst organisms (Chen et al., 2011; Beeby et al., 2016) and the F_o_ ATP synthase varies across taxa (Nirody et al., 2020). The F_o_ rotor c-ring stoichiometry ranges from eight subunits in mammalian mitochondrion (Zhou et al., 2015) to 17 in the bacterium *Burkholderia pseudomallei* (Schulz et al., 2017). Following this trend of organism-based modifications of molecular machines, three proteins described below interact with the T9SS of *P. gingivalis* but they are not essential for the functioning of the *F. johnsoniae* T9SS.

*PorU (Fjoh_1556/PGN_0022)* PorU of *P. gingivalis* is a part of the attachment complex that also involves PorZ, PorV, and PorQ. This complex modifies the T9SS substrates with an anionic lipopolysaccharide (A-LPS) and attaches them to the cell surface (Gorasia et al., 2015; Glew et al., 2017). PorU is a sortase which cleaves the CTD signal of the T9SS substrate and attaches the newly generated CTD of T9SS substrate to the cell surface *via* A-LPS (Glew et al., 2012, 2017). *P. gingivalis* cells lacking PorU have a partial T9SS defect and gingipain secretion is reduced (Glew et al., 2012). However, the *F. johnsoniae* PorU is not required for secretion of ChiA, RemA, or SprB, indicating that it does not play an essential role in the secretion of proteins by T9SS of *F. johnsoniae* (Kharade and McBride, 2015).

*PorZ (Fjoh_0707/PGN_0509) and PorQ (Fjoh_2755/PGN_0645)* PorZ of *P. gingivalis* interacts with A-LPS and PorU. Hence, it may play a role in providing A-LPS substrate to PorU (Madej et al., 2021). *P. gingivalis* cells lacking PorZ are defective in gingipain secretion (Lasica et al., 2016). PorQ is required for the association of PorZ with the outer membrane (Lasica et al., 2016). *F. johnsoniae* does not need PorQ (Fjoh_2755) for either gliding motility or chitin utilization (Shrivastava, 2013). No *porZ* mutant of *F. johnsoniae* has been reported thus far.

### Type 9 Secretion System Driven Swarm Behavior of Flavobacteria

**FIGURE 3:**
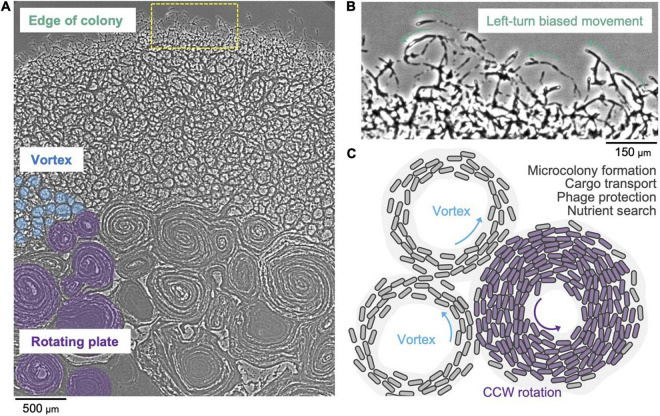
Collective motion of gliding *F. johnsoniae*. **(A)** Colony spreading pattern in a starved environment. **(B)** Left-turn biased cell movement at the edge of the colony. **(C)** Schematic of the vortex pattern formation and its possible roles.

It has long been known that *F. johnsoniae* forms spreading colonies on agar plates (Wolkin and Pate, 1984; Gorski et al., 1993). A recent study showed that the cells at high density are connected with one another and that in a starved environment they move in counterclockwise trajectories (Nakane et al., 2021). A *F. johnsoniae* swarm exhibits a vortex pattern that spontaneously appears as a lattice that is integrated into a large-scale circular plate (Figure 3A). Notably, the rotational direction of the circular plate is counterclockwise (Figure 3B). This two-dimensional pattern possibly appears due to the collision of cells and it further induces a nematic alignment of dense cells (Sumino et al., 2012). It is hypothesized that swarm behavior of T9SS driven Bacteroidetes may be involved in survival strategies and the efficient search of nutrients (Figure 3C; Nakane et al., 2021). A three-dimensional spherical microcolony was recently studied in a microfluidic device and it was reported that this biofilm-like microcolony self-assembles by the gliding motility (Li et al., 2021). It has also been reported that gliding motility contributes to the robustness of a *F. johnsoniae* biofilm (Eckroat et al., 2021). The colony of *Flavobacterium* strain Iridescent 1 (IR1) displays a bright brilliant green structural coloration, which is caused by the 2D close-packed cell arrangement aided by gliding motility (Johansen et al., 2018; Hamidjaja et al., 2020). This may provide photoprotection to either bacteria or host. Additionally, this structure might lead to the optimum cellular organization for the degradation of biological polymers (Johansen et al., 2018). The role of the structural coloration of the colony in the natural environment needs to be studied further. It is evident that our understanding of biofilm formation of *F. johnsoniae* and its dependence on swarm behavior is currently in its nascent stage. Future investigations in this area might reveal details about bacterial cooperation and behavior.

### Benefits of Swarm Behavior to the Microbiota

Type 9 secretion systems are found in bacterial genera that are abundant in the human oral and gut microbiota. Examples include the genera *Prevotella*, *Bacteroides*, *Parabacteroides*, *Capnocytophaga*, *Tanerella*, and *Porphyromonas*. *Capnocytophaga* sp. exhibit robust gliding motility (Holt et al., 1979; Leadbetter et al., 1979; Shrivastava et al., 2018) and are found in abundance in the oral microbiota. They are prominent members of human supra-gingival and sub-gingival biofilms (Mark Welch et al., 2016). In contrast with the wild-type strains isolated from the human oral microbiota, cells of *C. ochracea* that lack T9SS are non-motile and are deficient for biofilm formation (Kita et al., 2016).

*Capnocytophaga gingivalis* swarm in a multilayered counter clockwise vortex pattern (Shrivastava et al., 2018). *C. gingivalis* cells transport non-motile bacteria as cargo and shape the spatial organization of a polymicrobial community (Shrivastava et al., 2018). A *C. gingivalis* swarm can also transport bacteriophages as cargo (Ratheesh et al., 2021) and can increase the kinetics of death of a colony of prey bacteria. *Via* a combination of vortexing, layering, transportation of other bacteria, and phage transportation, T9SS-driven microbes play a crucial role in shaping a polymicrobial community. This amazing molecular machinery holds many secrets, which once unraveled, can significantly enrich our understanding of microbiology.

## Author Contributions

AT and AS contributed all sections besides the ones described below and created Figure 2. JG and AS contributed the section on diversity of protein secretion systems and created Figure 1. DN contributed the section on swarm behavior of Flavobacteria and created Figure 3. All authors contributed to the article and approved the submitted version.

## Conflict of Interest

The authors declare that the research was conducted in the absence of any commercial or financial relationships that could be construed as a potential conflict of interest.

## Publisher’s Note

All claims expressed in this article are solely those of the authors and do not necessarily represent those of their affiliated organizations, or those of the publisher, the editors and the reviewers. Any product that may be evaluated in this article, or claim that may be made by its manufacturer, is not guaranteed or endorsed by the publisher.

## References

[B1] Abrusci P. Vergara-Irigaray M. Johnson S. Beeby M. D. Hendrixson D. R. Roversi P. (2013). Architecture of the major component of the type III secretion system export apparatus. *Nat. Struct. Mol. Biol.* 20 99–104. 10.1038/nsmb.2452 23222644 PMC3537844

[B2] Agarwal S. Hunnicutt D. W. McBride M. J. (1997). Cloning and characterization of the *Flavobacterium johnsoniae* (*Cytophaga* johnsonae) gliding motility gene, gldA. *Proc. Natl. Acad. Sci. U.S.A.* 94 12139–12144. 10.1073/pnas.94.22.12139 9342376 PMC23729

[B3] Beckham K. S. H. Ritter C. Chojnowski G. Ziemianowicz D. S. Mullapudi E. Rettel M. (2021). Structure of the mycobacterial ESX-5 type VII secretion system pore complex. *Sci. Adv.* 7:eabg9923. 10.1126/sciadv.abg9923 34172453 PMC8232910

[B4] Beeby M. Ribardo D. A. Brennan C. A. Ruby E. G. Jensen G. J. Hendrixson D. R. (2016). Diverse high-torque bacterial flagellar motors assemble wider stator rings using a conserved protein scaffold. *Proc. Natl. Acad. Sci. U.S.A.* 113 E1917–E1926. 10.1073/pnas.1518952113 26976588 PMC4822576

[B5] Bhoite S. van Gerven N. Chapman M. R. Remaut H. (2019). Curli biogenesis: bacterial amyloid assembly by the type VIII secretion pathway. *Ecosal Plus* 8 ecosalplus.ESP-0037-2018. 10.1128/ecosalplus.ESP-0037-2018 30892177 PMC6428212

[B6] Blum M. Chang H.-Y. Chuguransky S. Grego T. Kandasaamy S. Mitchell A. (2021). The InterPro protein families and domains database: 20 years on. *Nucleic Acids Res.* 49 D344–D354. 10.1093/nar/gkaa977 33156333 PMC7778928

[B7] Braun T. F. McBride M. J. (2005). Flavobacterium johnsoniae GldJ is a lipoprotein that is required for gliding motility. *J. Bacteriol.* 187 2628–2637. 10.1128/JB.187.8.2628-2637.2005 15805509 PMC1070399

[B8] Braun T. F. Khubbar M. K. Saffarini D. A. McBride M. J. (2005). Flavobacterium johnsoniae gliding motility genes identified by mariner mutagenesis. *J. Bacteriol.* 187 6943–6952. 10.1128/JB.187.20.6943-6952.2005 16199564 PMC1251627

[B9] Bunduc C. M. Bitter W. Houben E. N. G. (2020). Structure and function of the mycobacterial type VII secretion systems. *Annu. Rev. Microbiol.* 74 315–335. 10.1146/annurev-micro-012420-081657 32660388

[B10] Chen S. Beeby M. Murphy G. E. Leadbetter J. R. Hendrixson D. R. Briegel A. (2011). Structural diversity of bacterial flagellar motors: structural diversity of bacterial flagellar motors. *EMBO J.* 30 2972–2981. 10.1038/emboj.2011.186 21673657 PMC3160247

[B11] Cianfanelli F. R. Monlezun L. Coulthurst S. J. (2016). Aim, load, fire: the type vi secretion system, a bacterial nanoweapon. *Trends Microbiol.* 24 51–62. 10.1016/j.tim.2015.10.005 26549582

[B12] Coulthurst S. (2019). The type VI secretion system: a versatile bacterial weapon. *Microbiology* 165 503–515. 10.1099/mic.0.000789 30893029

[B13] Craig L. Forest K. T. Maier B. (2019). Type IV pili: dynamics, biophysics and functional consequences. *Nat. Rev. Microbiol.* 17 429–440. 10.1038/s41579-019-0195-4 30988511

[B14] de Diego I. Ksiazek M. Mizgalska D. Koneru L. Golik P. Szmigielski B. (2016). The outer-membrane export signal of *Porphyromonas gingivalis* type IX secretion system (T9SS) is a conserved C-terminal β-sandwich domain. *Sci. Rep.* 6:23123. 10.1038/srep23123 27005013 PMC4804311

[B15] Denks K. Vogt A. Sachelaru I. Petriman N.-A. Kudva R. Koch H.-G. (2014). The Sec translocon mediated protein transport in prokaryotes and eukaryotes. *Mol. Membr. Biol.* 31 58–84. 10.3109/09687688.2014.907455 24762201

[B16] Diepold A. Armitage J. P. (2015). Type III secretion systems: the bacterial flagellum and the injectisome. *Philos. Trans. R. Soc. Lond. B. Biol. Sci.* 370:20150020. 10.1098/rstb.2015.0020 26370933 PMC4632597

[B17] Dunyak B. M. Gestwicki J. E. (2016). Peptidyl-Proline Isomerases (PPIases): targets for natural products and natural product-inspired compounds. *J. Med. Chem.* 59 9622–9644. 10.1021/acs.jmedchem.6b00411 27409354 PMC5501181

[B18] Eckroat T. J. Greguske C. Hunnicutt D. W. (2021). The type 9 secretion system is required for *Flavobacterium johnsoniae* biofilm formation. *Front. Microbiol.* 12:660887. 10.3389/fmicb.2021.660887 34539591 PMC8444969

[B19] Fan E. Chauhan N. Udatha D. B. R. K. G. Leo J. C. Linke D. (2016). Type V secretion systems in bacteria. *Microbiol. Spectr.* 4. 10.1128/microbiolspec.VMBF-0009-2015 26999388

[B20] Glew M. D. Veith P. D. Chen D. Gorasia D. G. Peng B. Reynolds E. C. (2017). PorV is an outer membrane shuttle protein for the type IX secretion system. *Sci. Rep.* 7:8790. 10.1038/s41598-017-09412-w 28821836 PMC5562754

[B21] Glew M. D. Veith P. D. Peng B. Chen Y.-Y. Gorasia D. G. Yang Q. (2012). PG0026 is the C-terminal signal peptidase of a novel secretion system of *Porphyromonas gingivalis*. *J. Biol. Chem.* 287 24605–24617. 10.1074/jbc.M112.369223 22593568 PMC3397888

[B22] Gómez-Santos N. Glatter T. Koebnik R. Świątek-Połatyńska M. A. Søgaard-Andersen L. (2019). A TonB-dependent transporter is required for secretion of protease PopC across the bacterial outer membrane. *Nat. Commun.* 10:1360. 10.1038/s41467-019-09366-9 30911012 PMC6434023

[B23] Gorasia D. G. Glew M. D. Veith P. D. Reynolds E. C. (2020). Quantitative proteomic analysis of the type IX secretion system mutants in *Porphyromonas gingivalis*. *Mol. Oral Microbiol.* 35 78–84. 10.1111/omi.12283 32040252

[B24] Gorasia D. G. Lunar Silva I. Butler C. A. Chabalier M. Doan T. Cascales E. (2022). Protein interactome analysis of the type IX secretion system identifies PorW as the missing link between the PorK/N ring complex and the Sov Translocon. *Microbiol. Spectr.* 10:e0160221. 10.1128/spectrum.01602-21 35019767 PMC8754138

[B25] Gorasia D. G. Veith P. D. Chen D. Seers C. A. Mitchell H. A. Chen Y.-Y. (2015). *Porphyromonas gingivalis* type IX secretion substrates are cleaved and modified by a sortase-like mechanism. *PLoS Pathog.* 11:e1005152. 10.1371/journal.ppat.1005152 26340749 PMC4560394

[B26] Gorasia D. G. Veith P. D. Hanssen E. G. Glew M. D. Sato K. Yukitake H. (2016). Structural insights into the PorK and PorN components of the *Porphyromonas gingivalis* type IX secretion system. *PLoS Pathog.* 12:e1005820. 10.1371/journal.ppat.1005820 27509186 PMC4980022

[B27] Gorasia D. Chreifi G. Seers C. Butler C. Heath J. Glew M. (2020). *In situ* structure and organisation of the type IX secretion system. *bioRxiv* [Preprint]. 10.1101/2020.05.13.094771

[B28] Gorski L. Godchaux W. Leadbetter E. R. (1993). Structural specificity of sugars that inhibit gliding motility of *Cytophaga* johnsonae. *Arch. Microbiol.* 160 121–125. 10.1007/BF00288713

[B29] Grossman A. S. Mauer T. J. Forest K. T. Goodrich-Blair H. (2021). A widespread bacterial secretion system with diverse substrates. *mBio* 12:e0195621. 10.1128/mBio.01956-21 34399622 PMC8406197

[B30] Hamidjaja R. Capoulade J. Catón L. Ingham C. J. (2020). The cell organization underlying structural colour is involved in Flavobacterium IR1 predation. *ISME J.* 14 2890–2900. 10.1038/s41396-020-00760-6 32873891 PMC7784876

[B31] Heath J. E. Seers C. A. Veith P. D. Butler C. A. Nor Muhammad N. A. Chen Y.-Y. (2016). PG1058 is a novel multidomain protein component of the bacterial type IX secretion system. *PLoS One* 11:e0164313. 10.1371/journal.pone.0164313 27711252 PMC5053529

[B32] Hennell James R. Deme J. C. Kjær A. Alcock F. Silale A. Lauber F. (2021). Structure and mechanism of the proton-driven motor that powers type 9 secretion and gliding motility. *Nat. Microbiol.* 6 221–233. 10.1038/s41564-020-00823-6 33432152 PMC7116788

[B33] Hennon S. W. Soman R. Zhu L. Dalbey R. E. (2015). YidC/Alb3/Oxa1 family of insertases. *J. Biol. Chem.* 290 14866–14874. 10.1074/jbc.R115.638171 25947384 PMC4463434

[B34] Hofreuter D. Odenbreit S. Haas R. (2001). Natural transformation competence in *Helicobacter pylori* is mediated by the basic components of a type IV secretion system: H. pylori natural transformation competence. *Mol. Microbiol.* 41 379–391. 10.1046/j.1365-2958.2001.02502.x 11489125

[B35] Holt S. C. Leadbetter E. R. Socransky S. S. (1979). Capno*cytophaga*: new genus of gram-negative gliding bacteria. II. Morphology and ultrastructure. *Arch. Microbiol.* 122 17–27. 10.1007/BF00408041 518235

[B36] Hunnicutt D. W. McBride M. J. (2000). Cloning and characterization of the *Flavobacterium johnsoniae* gliding-motility genes gldB and gldC. *J. Bacteriol.* 182 911–918. 10.1128/JB.182.4.911-918.2000 10648514 PMC94364

[B37] Hunnicutt D. W. McBride M. J. (2001). Cloning and characterization of the *Flavobacterium johnsoniae* gliding motility genes gldD and gldE. *J. Bacteriol.* 183 4167–4175. 10.1128/JB.183.14.4167-4175.2001 11418556 PMC95305

[B38] Hunnicutt D. W. Kempf M. J. McBride M. J. (2002). Mutations in *Flavobacterium johnsoniae* gldF and gldG disrupt gliding motility and interfere with membrane localization of GldA. *J. Bacteriol.* 184 2370–2378. 10.1128/JB.184.9.2370-2378.2002 11948149 PMC134979

[B39] Johansen V. E. Catón L. Hamidjaja R. Oosterink E. Wilts B. D. Rasmussen T. S. (2018). Genetic manipulation of structural color in bacterial colonies. *Proc. Natl. Acad. Sci. U.S.A.* 115 2652–2657. 10.1073/pnas.1716214115 29472451 PMC5856530

[B40] Johnston J. J. Shrivastava A. McBride M. J. (2018). Untangling *Flavobacterium johnsoniae* gliding motility and protein secretion. *J. Bacteriol.* 200 e00362-17. 10.1128/JB.00362-17 29109184 PMC5738736

[B41] Kharade S. S. McBride M. J. (2014). *Flavobacterium johnsoniae* chitinase ChiA is required for chitin utilization and is secreted by the type IX secretion system. *J. Bacteriol.* 196 961–970. 10.1128/JB.01170-13 24363341 PMC3957688

[B42] Kharade S. S. McBride M. J. (2015). *Flavobacterium johnsoniae* PorV is required for secretion of a subset of proteins targeted to the type IX secretion system. *J. Bacteriol.* 197 147–158. 10.1128/JB.02085-14 25331433 PMC4288674

[B43] Kita D. Shibata S. Kikuchi Y. Kokubu E. Nakayama K. Saito A. (2016). Involvement of the Type IX secretion system in *Capnocytophaga ochracea* gliding motility and biofilm formation. *Appl. Environ. Microbiol.* 82 1756–1766. 10.1128/AEM.03452-15 26729712 PMC4784043

[B44] Korotkov K. V. Sandkvist M. (2019). Architecture, function, and substrates of the Type II secretion system. *EcoSal Plus* 8 ecosallus.ES–0034–2018. 10.1128/ecosalplus.ESP-0034-2018 30767847 PMC6638579

[B45] Kudva R. Denks K. Kuhn P. Vogt A. Müller M. Koch H.-G. (2013). Protein translocation across the inner membrane of Gram-negative bacteria: the Sec and Tat dependent protein transport pathways. *Res. Microbiol.* 164 505–534. 10.1016/j.resmic.2013.03.016 23567322

[B46] Kulkarni S. S. Johnston J. J. Zhu Y. Hying Z. T. McBride M. J. (2019). The carboxy-terminal region of *Flavobacterium johnsoniae* SprB facilitates its secretion by the type IX secretion system and propulsion by the gliding motility machinery. *J. Bacteriol.* 201 e218–e219. 10.1128/JB.00218-19 31262839 PMC6755757

[B47] Kulkarni S. S. Zhu Y. Brendel C. J. McBride M. J. (2017). Diverse C-terminal sequences involved in *Flavobacterium johnsoniae* protein secretion. *J. Bacteriol.* 199 e00884–16. 10.1128/JB.00884-16 28396348 PMC5446621

[B48] Kusters I. Driessen A. J. M. (2011). SecA, a remarkable nanomachine. *Cell. Mol. Life Sci.* 68 2053–2066. 10.1007/s00018-011-0681-y 21479870 PMC3101351

[B49] Lasica A. M. Goulas T. Mizgalska D. Zhou X. de Diego I. Ksiazek M. (2016). Structural and functional probing of PorZ, an essential bacterial surface component of the type-IX secretion system of human oral-microbiomic Porphyromonas gingivalis. *Sci. Rep.* 6:37708. 10.1038/srep37708 27883039 PMC5121618

[B50] Lauber F. Deme J. C. Lea S. M. Berks B. C. (2018). Type 9 secretion system structures reveal a new protein transport mechanism. *Nature* 564 77–82. 10.1038/s41586-018-0693-y 30405243 PMC6927815

[B51] Leadbetter E. R. Holt S. C. Socransky S. S. (1979). Capno*cytophaga*: new genus of gram-negative gliding bacteria. I. General characteristics, taxonomic considerations and significance. *Arch. Microbiol.* 122 9–16. 10.1007/BF00408040 518239

[B52] Leone P. Roche J. Vincent M. S. Tran Q. H. Desmyter A. Cascales E. (2018). Type IX secretion system PorM and gliding machinery GldM form arches spanning the periplasmic space. *Nat. Commun.* 9:429. 10.1038/s41467-017-02784-7 29382829 PMC5790014

[B53] Li C. Hurley A. Hu W. Warrick J. W. Lozano G. L. Ayuso J. M. (2021). Social motility of biofilm-like microcolonies in a gliding bacterium. *Nat. Commun.* 12:5700. 10.1038/s41467-021-25408-7 34588437 PMC8481357

[B54] Li Y. G. Hu B. Christie P. J. (2019). Biological and structural diversity of type IV secretion systems. *Microbiol. Spectr.* 7. 10.1128/microbiolspec.PSIB-0012-2018 30953428 PMC6452883

[B55] Madej M. Nowakowska Z. Ksiazek M. Lasica A. M. Mizgalska D. Nowak M. (2021). PorZ, an essential component of the type IX secretion system of *Porphyromonas gingivalis*, delivers anionic lipopolysaccharide to the PorU sortase for transpeptidase processing of T9SS cargo proteins. *mBio* 12 e02262–20. 10.1128/mBio.02262-20 33622730 PMC8545088

[B56] Mark Welch J. L. Rossetti B. J. Rieken C. W. Dewhirst F. E. Borisy G. G. (2016). Biogeography of a human oral microbiome at the micron scale. *Proc. Natl. Acad. Sci. U.S.A.* 113 E791–E800. 10.1073/pnas.1522149113 26811460 PMC4760785

[B57] McBride M. J. Braun T. F. (2004). GldI is a lipoprotein that is required for *Flavobacterium johnsoniae* gliding motility and chitin utilization. *J. Bacteriol.* 186 2295–2302. 10.1128/JB.186.8.2295-2302.2004 15060031 PMC412174

[B58] McBride M. J. Zhu Y. (2013). Gliding motility and Por secretion system genes are widespread among members of the phylum bacteroidetes. *J. Bacteriol.* 195 270–278. 10.1128/JB.01962-12 23123910 PMC3553832

[B59] McBride M. J. Braun T. F. Brust J. L. (2003). *Flavobacterium johnsoniae* GldH is a lipoprotein that is required for gliding motility and chitin utilization. *J. Bacteriol.* 185 6648–6657. 10.1128/JB.185.22.6648-6657.2003 14594839 PMC262120

[B60] Meuskens I. Saragliadis A. Leo J. C. Linke D. (2019). Type V secretion systems: an overview of passenger domain functions. *Front. Microbiol.* 10:1163. 10.3389/fmicb.2019.01163 31214135 PMC6555100

[B61] Nakane D. Odaka S. Suzuki K. Nishizaka T. (2021). Large-Scale vortices with dynamic rotation emerged from monolayer collective motion of gliding Flavobacteria. *J. Bacteriol.* 203:e0007321. 10.1128/JB.00073-21 33927052 PMC8223929

[B62] Nakane D. Sato K. Wada H. McBride M. J. Nakayama K. (2013). Helical flow of surface protein required for bacterial gliding motility. *Proc. Natl. Acad. Sci. U.S.A.* 110 11145–11150. 10.1073/pnas.1219753110 23781102 PMC3704026

[B63] Naskar S. Hohl M. Tassinari M. Low H. H. (2021). The structure and mechanism of the bacterial type II secretion system. *Mol. Microbiol.* 115 412–424. 10.1111/mmi.14664 33283907

[B64] Nelson S. S. Bollampalli S. McBride M. J. (2008). SprB is a cell surface component of the *Flavobacterium johnsoniae* gliding motility machinery. *J. Bacteriol.* 190 2851–2857. 10.1128/JB.01904-07 18281397 PMC2293251

[B65] Nguyen K.-A. Żylicz J. Szczesny P. Sroka A. Hunter N. Potempa J. (2009). Verification of a topology model of PorT as an integral outer-membrane protein in *Porphyromonas gingivalis*. *Microbiol. Read. Engl.* 155 328–337. 10.1099/mic.0.024323-0 19202082 PMC2729710

[B66] Nirody J. A. Budin I. Rangamani P. (2020). ATP synthase: evolution, energetics, and membrane interactions. *J. Gen. Physiol.* 152:e201912475. 10.1085/jgp.201912475 32966553 PMC7594442

[B67] Palmer T. Berks B. C. (2012). The twin-arginine translocation (Tat) protein export pathway. *Nat. Rev. Microbiol.* 10 483–496. 10.1038/nrmicro2814 22683878

[B68] Palmer T. Finney A. J. Saha C. K. Atkinson G. C. Sargent F. (2021). A holin/peptidoglycan hydrolase-dependent protein secretion system. *Mol. Microbiol.* 115 345–355. 10.1111/mmi.14599 32885520

[B69] Papanikou E. Karamanou S. Economou A. (2007). Bacterial protein secretion through the translocase nanomachine. *Nat. Rev. Microbiol.* 5 839–851. 10.1038/nrmicro1771 17938627

[B70] Portaliou A. G. Tsolis K. C. Loos M. S. Zorzini V. Economou A. (2016). Type III secretion: building and operating a remarkable nanomachine. *Trends Biochem. Sci.* 41 175–189. 10.1016/j.tibs.2015.09.005 26520801

[B71] Ratheesh N. K. Calderon C. A. Zdimal A. M. Shrivastava A. (2021). Bacterial swarm-mediated phage transportation disrupts a biofilm inherently protected from phage penetration. *bioRxiv* [Preprint]. 10.1101/2021.06.25.449910 PMC10434198 37358420

[B72] Rhodes R. G. Samarasam M. N. Shrivastava A. van Baaren J. M. Pochiraju S. Bollampalli S. (2010). *Flavobacterium johnsoniae* gldN and gldO are partially redundant genes required for gliding motility and surface localization of SprB. *J. Bacteriol.* 192 1201–1211. 10.1128/JB.01495-09 20038590 PMC2820869

[B73] Rhodes R. G. Samarasam M. N. Van Groll E. J. McBride M. J. (2011). Mutations in *Flavobacterium johnsoniae* sprE result in defects in gliding motility and protein secretion. *J. Bacteriol.* 193 5322–5327. 10.1128/JB.05480-11 21784937 PMC3187464

[B74] Rivera-Calzada A. Famelis N. Llorca O. Geibel S. (2021). Type VII secretion systems: structure, functions and transport models. *Nat. Rev. Microbiol.* 19 567–584. 10.1038/s41579-021-00560-5 34040228

[B75] Saiki K. Konishi K. (2007). Identification of a *Porphyromonas gingivalis* novel protein sov required for the secretion of gingipains. *Microbiol. Immunol.* 51 483–491. 10.1111/j.1348-0421.2007.tb03936.x 17579257

[B76] Saiki K. Konishi K. (2010). The role of Sov protein in the secretion of gingipain protease virulence factors of *Porphyromonas gingivalis*. *FEMS Microbiol. Lett.* 302 166–174. 10.1111/j.1574-6968.2009.01848.x 20002184

[B77] Sato K. Naito M. Yukitake H. Hirakawa H. Shoji M. McBride M. J. (2010). A protein secretion system linked to bacteroidete gliding motility and pathogenesis. *Proc. Natl. Acad. Sci. U.S.A.* 107 276–281. 10.1073/pnas.0912010107 19966289 PMC2806738

[B78] Sato K. Sakai E. Veith P. D. Shoji M. Kikuchi Y. Yukitake H. (2005). Identification of a new membrane-associated protein that influences transport/maturation of gingipains and adhesins of *Porphyromonas gingivalis*. *J. Biol. Chem.* 280 8668–8677. 10.1074/jbc.M413544200 15634642

[B79] Sato K. Yukitake H. Narita Y. Shoji M. Naito M. Nakayama K. (2013). Identification of *Porphyromonas gingivalis* proteins secreted by the Por secretion system. *FEMS Microbiol. Lett.* 338 68–76. 10.1111/1574-6968.12028 23075153

[B80] Schulz S. Wilkes M. Mills D. J. Kühlbrandt W. Meier T. (2017). Molecular architecture of the N-type ATPase rotor ring from Burkholderia pseudomallei. *EMBO Rep.* 18 526–535. 10.15252/embr.201643374 28283532 PMC5376962

[B81] Shrivastava A. K. (2013). *Cell Surface Adhesins, Exopolysaccharides and the Por (Type IX) Secretion System of Flavobacterium johnsoniae. Theses and Dissertations.* Milwaukee, WI: University of Wisconsin-Milwaukee.

[B82] Shrivastava A. Berg H. C. (2020). A molecular rack and pinion actuates a cell-surface adhesin and enables bacterial gliding motility. *Sci. Adv.* 6:eaay6616. 10.1126/sciadv.aay6616 32181348 PMC7056307

[B83] Shrivastava A. Johnston J. J. van Baaren J. M. McBride M. J. (2013). Flavobacterium johnsoniae GldK, GldL, GldM, and SprA are required for secretion of the cell surface gliding motility adhesins SprB and RemA. *J. Bacteriol.* 195 3201–3212. 10.1128/JB.00333-13 23667240 PMC3697645

[B84] Shrivastava A. Lele P. P. Berg H. C. (2015). A rotary motor drives Flavobacterium gliding. *Curr. Biol.* 25 338–341. 10.1016/j.cub.2014.11.045 25619763 PMC4319542

[B85] Shrivastava A. Patel V. K. Tang Y. Yost S. C. Dewhirst F. E. Berg H. C. (2018). Cargo transport shapes the spatial organization of a microbial community. *Proc. Natl. Acad. Sci. U.S.A.* 115 8633–8638. 10.1073/pnas.1808966115 30082394 PMC6112710

[B86] Shrivastava A. Rhodes R. G. Pochiraju S. Nakane D. McBride M. J. (2012). *Flavobacterium johnsoniae* RemA is a mobile cell surface lectin involved in gliding. *J. Bacteriol.* 194 3678–3688. 10.1128/JB.00588-12 22582276 PMC3393505

[B87] Shrivastava A. Roland T. Berg H. C. (2016). The screw-like movement of a gliding bacterium is powered by spiral motion of cell-surface adhesins. *Biophys. J.* 111 1008–1013. 10.1016/j.bpj.2016.07.043 27602728 PMC5018149

[B88] Slakeski N. Seers C. A. Ng K. Moore C. Cleal S. M. Veith P. D. (2011). C-Terminal domain residues important for secretion and attachment of RgpB in *Porphyromonas gingivalis*. *J. Bacteriol.* 193 132–142. 10.1128/JB.00773-10 20971915 PMC3019955

[B89] Spirig T. Weiner E. M. Clubb R. T. (2011). Sortase enzymes in Gram-positive bacteria: sortase enzymes in Gram-positive bacteria. *Mol. Microbiol.* 82 1044–1059. 10.1111/j.1365-2958.2011.07887.x 22026821 PMC3590066

[B90] Spitz O. Erenburg I. N. Beer T. Kanonenberg K. Holland I. B. Schmitt L. (2019). Type I secretion systems—one mechanism for all? *Microbiol. Spectr.* 7. 10.1128/microbiolspec.PSIB-0003-2018 30848237 PMC11588160

[B91] Sumino Y. Nagai K. H. Shitaka Y. Tanaka D. Yoshikawa K. Chaté H. (2012). Large-scale vortex lattice emerging from collectively moving microtubules. *Nature* 483 448–452. 10.1038/nature10874 22437613

[B92] Trinh N. T. T. Tran H. Q. Van Dong Q. Cambillau C. Roussel A. Leone P. (2020). Crystal structure of Type IX secretion system PorE C-terminal domain from *Porphyromonas gingivalis* in complex with a peptidoglycan fragment. *Sci. Rep.* 10:7384. 10.1038/s41598-020-64115-z 32355178 PMC7192894

[B93] Van Gerven N. Klein R. D. Hultgren S. J. Remaut H. (2015). Bacterial amyloid formation: structural insights into curli biogensis. *Trends Microbiol.* 23 693–706. 10.1016/j.tim.2015.07.010 26439293 PMC4636965

[B94] Vincent M. S. Canestrari M. J. Leone P. Stathopulos J. Ize B. Zoued A. (2017). Characterization of the *Porphyromonas gingivalis* type IX secretion trans-envelope PorKLMNP core complex. *J. Biol. Chem.* 292 3252–3261. 10.1074/jbc.M116.765081 28057754 PMC5336160

[B95] Vincent M. S. Hervada C. C. Sebban-Kreuzer C. Guenno H. L. Chabalier M. Kosta A. (2021). Dynamic proton-dependent motors power type IX secretion and gliding adhesin movement in Flavobacterium. *bioRxiv* [Preprint]. 10.1101/2021.10.19.464928 PMC8986121 35333857

[B96] Vorburger T. Stein A. Ziegler U. Kaim G. Steuber J. (2009). Functional role of a conserved aspartic acid residue in the motor of the Na(+)-driven flagellum from *Vibrio cholerae*. *Biochim. Biophys. Acta* 1787 1198–1204. 10.1016/j.bbabio.2009.05.015 19501041

[B97] Waksman G. Hultgren S. J. (2009). Structural biology of the chaperone–usher pathway of pilus biogenesis. *Nat. Rev. Microbiol.* 7 765–774. 10.1038/nrmicro2220 19820722 PMC3790644

[B98] Wolkin R. H. Pate J. L. (1984). Translocation of motile cells of the gliding bacterium *Cytophaga johnsonae* depends on a surface component that may be modified by sugars. *Microbiology* 130 2651–2669. 10.1099/00221287-130-10-2651

[B99] Yamaguchi T. Makino F. Miyata T. Minamino T. Kato T. Namba K. (2021). Structure of the molecular bushing of the bacterial flagellar motor. *Nat. Commun.* 12:4469. 10.1038/s41467-021-24715-3 34294704 PMC8298488

[B100] Zhou A. Rohou A. Schep D. G. Bason J. V. Montgomery M. G. Walker J. E. (2015). Structure and conformational states of the bovine mitochondrial ATP synthase by cryo-EM. *eLife* 4:e10180. 10.7554/eLife.10180 26439008 PMC4718723

[B101] Zhou J. Sharp L. L. Tang H. L. Lloyd S. A. Billings S. Braun T. F. (1998). Function of protonatable residues in the flagellar motor of *Escherichia coli*: a critical role for Asp 32 of MotB. *J. Bacteriol.* 180 2729–2735. 10.1128/JB.180.10.2729-2735.1998 9573160 PMC107227

[B102] Zhu Y. McBride M. J. (2014). Deletion of the *Cytophaga hutchinsonii* type IX secretion system gene sprP results in defects in gliding motility and cellulose utilization. *Appl. Microbiol. Biotechnol.* 98 763–775. 10.1007/s00253-013-5355-2 24257839

